# Effects of Different Dietary Levels of Blue Lupine (*Lupinus angustifolius*) Seed Meal With or Without Probiotics on the Performance, Carcass Criteria, Immune Organs, and Gut Morphology of Broiler Chickens

**DOI:** 10.3389/fvets.2020.00124

**Published:** 2020-03-13

**Authors:** Ahmed A. Al-Sagan, Abdullah H. AL-Yemni, Abdulaziz A. Al-Abdullatif, Youssef A. Attia, Elsayed O. S Hussein

**Affiliations:** ^1^King Abdulaziz City for Science and Technology, Riyadh, Saudi Arabia; ^2^Arabian Agriculture Services Company (ARASCO), Riyadh, Saudi Arabia; ^3^Department of Animal Production, College of Food and Agriculture Sciences, King Saud University, Riyadh, Saudi Arabia; ^4^Department of Arid Land Agriculture, College of Meteorology, Environment and Arid Land Agriculture, King Abdualziz University, Jeddah, Saudi Arabia

**Keywords:** blue lupine, probiotics, broilers, carcass quality, intestinal morphology, immune organs

## Abstract

This study aimed to investigate the effects of different dietary levels of blue lupine (*Lupinus angustifolius*) seed meal with or without probiotics (*Bacillus subtilis*) in broiler diets on the growth performance, carcass characteristics, internal and immune organs, and gut morphology. Three experimental diets containing 0, 20, and 30% of blue lupine, with or without probiotics, were formulated and fed to 144 day (d)-old Ross 308 broiler chickens. Overall, chicks fed blue lupine meal diets, especially at the 30% rate, showed improved growth, feed performance parameters, and carcass characteristics in comparison to chicks fed a soybean meal-based diet. For example, a 30% blue lupine diet resulted in a significant increase in the duodenum length percentage of 35 d-old broilers; the addition of probiotics had no—effects on the dressing, thigh, and leg percentages of 21- and 35 d-old broilers and the drumstick and leg percentages of 35 d-old broilers. In conclusion, a 30% blue lupine seed diet with the addition of probiotics could provide a cheap source of protein without negative effects on the growth performance, carcass characteristics, immune organs and gut morphology of broilers.

## Introduction

Poultry production necessitates the provision of adequate protein and amino acids to chicks for normal growth and maximum production (1). The protein requirements of broiler chick with adequate growth performance and body weight are high (2, 3). The cost of importing feed ingredients such as soybean meal and corn to formulate poultry diets is one of the most important impediments to achieving high productivity. Soybean meal, preferred for its high nutritional value, is the main source of protein for broilers. However, the soybean meal price fluctuates worldwide with a tendency to increase with decreasing supply during certain periods of the year (4). The increase in the cost of broiler diets is mainly due to the volatility of the feed market and the stiff competition for food resources between human and animal diets and the biofuel-producing industries (5, 6). Thus, there is a need for local feed resources that will provide alternatives to soybean meal, thereby decreasing feed costs.

One of the most important strategies for the poultry industry is to develop domestic dietary formulations, which will allow the use of local ingredients as substitutes for imported feed ingredients and, subsequently, reduce diet costs. Legume seeds such as the blue lupine (*Lupinus angustifolius*), which belongs to the family Fabaceae, are important sources of protein for monogastric animals and are considered as alternatives to the soybean meal (7, 8). Blue lupine is characterized by its year-round availability and its low price compared to soybean meal. The use of lupine seeds in the formulation of broiler diets is justified primarily because of their high protein content (**≈**40.08%) (9). Also, lupine is a good source of nutrients such as lipids, fiber, minerals, and vitamins after the ban of use soybean meal in organic farming (10–12).

The main anti-nutritional factors in lupins are alkaloids, phytates, protease inhibitors, and lectins, which cause retarded growth and poor feed utilization (13). Lupine has a high non-starch polysaccharide (NSP) content (14). The main NSP in lupine seeds is galactan that consists of different amounts of arabinose and galactose monosaccharides. NSPs impair gut ecology and reduce the digestibility of poultry diets (2, 15, 16). Reduced feed intake and growth rates are noted in birds that are fed lupine-based diets (2, 7); these adverse effects were noted during the first week and lasted up until the end of the experiments. However, lupine species such as *L. angustifolius* that have low-alkaloid content are increasingly used in both layers' and broilers' diets (17). Blue lupine can be incorporated in broilers' diets at 10% and yield similar results to the soybean meal diet, but the increasing level to 20% decreased performance while increasing wet droppings (18). Lupine flour can constitute up to 30% of soybean meal protein of broilers' diets while maintaining their production performance in good levels, but increasing blue lupine to 40% (18% in the diet) and 80% (~30% in the diet) of soybean meal protein decreased growth performance (19). Lupines can constitute up to 15% of layers' diets without any negative effects on their production performance and health (8). Thus, overcoming the anti-nutritional effects of lupine and improving the utilization of lupine NSPs require further research (20–22).

Probiotics are well-known microorganisms that have a positive effect on the performance of the host bird by improving the ecology of the gut (23–25). Growth performance and feed conversion rate (FCR) are improved in broiler chickens supplemented with probiotics (26–28). Probiotics improve gut ecology, immunity and eliminate toxic effects on animals (29–31). In literature, there were rare studies used probiotic as a tool to improve the use of blue lupine in chickens' feeding due to the negative effect of blue lupine on gut eco-system as evident by increasing wet dropping (18). Furthermore, the use of probiotics in the literature to improve animal performance and gut ecology has received great attention with some success (23–28). Thus, we hypothesized that probiotics supplementation to broilers' diets containing 30% blue lupine might improve growth performance and carcass traits due to improving gut ecosystem. Hence, the current study aimed to evaluate the effects of lupine (*Lupinus angustifolius* L. “Boltensia”) seed meal inclusion in broiler diets, with or without probiotics (*Bacillus subtilis*), on the performance, carcass quality, internal organs, immune system, and gut morphology of Ross 308 broiler chickens.

## Materials and Methods

### Diets, Probiotics, and Experimental Design

The *L. angustifolius* cultivar Boltensia, a low-alkaloid variety, was used in the present broiler study. Blue lupine seeds were milled in a hammer mill, sieved through a 3 mm screen, and mixed with the other ingredients. The chemical composition of blue lupine was determined according to (29) and used in diet formulation. The metabolizable energy value was calculated by using the equation published by (32):

$$\begin{array}{l} \text{AMEn}=\text{CP}\left(\text{g}/\text{kg}\right)\times 16.59+\text{fat}\left(\text{g}/\text{kg}\right)\times 33+1.559\times \text{NFE }\left(\text{g}/\text{kg}\right). \end{array}$$

AMEn = Nitrogen corrected metabolizable energy, CP = Crude protein, NFE = Nitrogen free extract (starch + sugar).

The composition of the experimental diets is presented in Tables 1, 2. Blue lupine was included in the experimental diets at 0, 20, and 30% with similar calorific and nitrogenous values. The experimental diets were formulated to meet or exceed the minimum broiler requirements (33). The broilers were fed diets with or without probiotics [the *Bacillus subtilis, PB6* based-probiotic that was used in this experiment was CS (CloSTAT® brand, Kemin Industries Inc., Des Moines, IA, USA). The commercially available product (product no. 017176) contains live viable ≥1 × 10^11^ cfu/g *B*. subtilis PB6 according to the manufacturer (1.5 g of the product to 1 ton of feed), https://www.kemin.com/na/en-us/products/clostat. Thus, the experimental design included 3 concentrations of blue lupine × 2 two levels of *B. subtilis* (0 and 0.05 g/kg diet) in a factorial arrangement. The probiotics were used as an ideal agent for improving gut ecology due to excepted negative effects of blue lupine in the gut ecosystem. The probiotic products were mixed with a small amount of corn in a small mixer before being transferred to a larger mixer with the remaining components of the diet, to ensure homogeneity. Feed-in a mashed form and water were available *ad libitum*.

**Table 1:** The composition of the diets used in the experiment (starter).

**Ingredients, %**	**1**	**2**	**3**	**4**	**5**	**6**
Blue lupine	0	0	20	20	30	30
Yellow corn	57.3	57.14985	47.2	47.04985	43.5	43.44985
Soybean meal 48% Crude protein	35.81	35.81	24.2	24.2	16.8	16.8
Palm oil	2.40	2.50	3.60	3.70	4.00	4.00
Dicalcium phosphate	1.74	1.74	1.87	1.87	1.96	1.96
Limestone	1.27	1.27	1.17	1.17	1.11	1.11
Salt	0.46	0.46	0.45	0.45	0.45	0.45
L-Threonine	0	0	0.15	0.15	0.25	0.25
DL-Methionine	0.19	0.19	0.36	0.36	0.46	0.46
L-Lysine hydrochloride	0.03	0.03	0.20	0.20	0.46	0.46
Choline Cl70	0.05	0.05	0.05	0.05	0.05	0.05
Minvit Arasco 0.5%^a^	0.5	0.5	0.5	0.5	0.5	0.5
Probiotic^b^	0	0.00015	0	0.00015	0	0.00015
Anti coccidial and mold	0.25	0.30	0.25	0.30	0.25	0.30
CreAmino	0	0	0	0	0.21	0.21
Total	100	100	100	100	100	100
**Nutrient compositions**						
Dry matter %	89.4	89.4	90.2	90.2	90.6	90.6
Metabolizable energy MJ/kg	12.6	12.6	12.6	12.6	12.6	12.6
Crude protein %	22.1	22.2	22.1	22.0	22.2	22.1
Arginine %	1.53	1.53	1.30	1.30	1.30	1.29
Lysine %	1.26	1.26	1.10	1.10	1.10	1.10
Methionine %	0.53	0.53	0.61	0.61	0.66	0.66
Cystine %	0.36	0.36	0.26	0.26	0.20	0.20
Methionine + cystine %	0.90	0.90	0.90	0.90	0.90	0.90
Threonine %	0.85	0.85	0.82	0.82	0.80	0.80
Tryptophan %	0.28	0.28	0.21	0.21	0.17	0.17
Valine %	1.03	1.03	0.80	0.80	0.66	0.66
Ether extract	4.94	5.02	6.72	6.79	7.39	7.46
Linoleic acid %	1.62	1.62	1.46	1.47	1.39	1.39
Crude Fiber	2.66	2.65	5.23	5.22	6.48	6.48
Calcium %	1.01	0.998	1.02	0.997	1.01	1.02
Available phosphorus %	0.45	0.45	0.45	0.45	0.45	0.45
Sodium %	0.20	0.20	0.20	0.20	0.20	0.20

a *Provided per kg diet: vit. A 24,000,000 IU/kg, vit. D3 10,00,000 IU/kg, vit. E 16,000 IU kg, vit. K3 800 mg/kg, vit. B1 600 mg/kg, vit. B2 1,600 mg/kg, vit. B6 1,000 mg/ kg, vit. B12 6 mg/kg, Biotin 40 mg/kg, Folic Acid 400 mg/kg, Niacin 8,000 mg/kg, pantothenic Acid 3,000 mg/kg, Antioxidant 3,000 mg/kg, Cobalt 80 mg/kg, Copper 2,000 mg/kg, Iodine 400 mg/kg, Iron 1,200 mg/kg, manganese 18,000 mg/kg, Selenium 60 mg/kg, Zinc 60 mg/kg*.

b *Probiotic (Bacillus subtilis) CloSTAT® brand*.

**Table 2:** The composition of the diets used in the experiment (finisher).

**Ingredients**	**1**	**2**	**3**	**4**	**5**	**6**
Blue lupine	0	0	20	20	30	30
Yellow corn	61.915	61.76485	51.945	51.77485	47.065	46.93485
Soybean meal 48% Crude protein	31.03	31.06	19.28	19.32	13.24	13.25
Palm oil	3.18	3.25	4.37	4.45	4.93	5.00
Dicalcium phosphate	1.24	1.24	1.37	1.37	1.44	1.44
Limestone	1.37	1.37	1.26	1.26	1.21	1.21
Salt	0.33	0.33	0.32	0.32	0.32	0.32
L-Threonine	0	0	0.18	0.18	0.23	0.23
DL-Methionine	0.06	0.06	0.23	0.23	0.31	0.31
L-Lysine hydrochloride	0.08	0.08	0.25	0.25	0.46	0.46
Choline Cl70	0.045	0.045	0.045	0.045	0.045	0.045
Minvit Arasco 0.5%^a^	0.5	0.5	0.5	0.5	0.5	0.5
Probiotic^b^	0	0.00015	0	0.00015	0	0.00015
Anti coccidial and mold	0.25	0.30	0.25	0.30	0.25	0.30
CreAmino	0	0	0	0	0	0
Total	100	100	100	100	100	100
**Nutrient compositions**						
Dry matter %	89.4	89.4	90.2	90.1	90.6	90.5
Metabolizable energy MJ/kg	13.0	13.0	13.0	13.0	13.0	13.0
Crude protein %	19.9	20.1	20.0	20.2	20.0	19.8
Arginine %	1.37	1.37	1.14	1.14	1.01	1.01
Lysine %	1.16	1.16	1.00	1.00	1.00	1.00
Methionine %	0.38	0.38	0.46	0.46	0.50	0.497
Cystine %	0.335	0.335	0.232	0.232	0.18	0.18
Methionine + cystine %	0.72	0.72	0.72	0.72	0.72	0.72
Threonine %	0.77	0.77	0.77	0.77	0.72	0.72
Tryptophan %	0.25	0.25	0.18	0.18	0.15	0.15
Valine %	0.94	0.94	0.71	0.71	0.60	0.60
Ether extract	5.84	5.91	7.62	7.68	8.47	8.53
Linoleic acid %	1.78	1.78	1.62	1.62	1.54	1.54
Crude Fiber	2.57	2.57	5.14	5.14	6.42	6.42
Calcium %	0.913	0.907	0.901	0.911	0.897	0.903
Available phosphorus %	0.35	0.35	0.35	0.35	0.35	0.35
Sodium %	0.15	0.15	0.15	0.15	0.15	0.15

a *Provided per kg diet: Vit. A 24,000,000 IU/kg, Vit. D3 10,00,000 IU/kg, Vit. E 16,000 IU kg, Vit. K3 800 mg/kg, Vit. B1 600 mg/kg, Vit. B2 1,600 mg/kg, Vit. B6 1,000 mg/ kg, Vit. B12 6 mg/kg, Biotin 40 mg/kg, Folic Acid 400 mg/kg, Niacin 8,000 mg/kg, pantothenic Acid 3,000 mg/kg, Antioxidant 3,000 mg/kg, Cobalt 80 mg/kg, Copper 2,000 mg/kg, Iodine 400 mg/kg, Iron 1,200 mg/kg, manganese 18,000 mg/kg, Selenium 60 mg/kg, Zinc 60 mg/kg*.

b *Probiotic (Bacillus subtilis) CloSTAT® brand*.

### Chickens, Housing, and Husbandry

A total of 144 unsexed, 1 d-old Ross 308 broiler chicks were obtained from the Al-Wadi Company and were distributed randomly to 24 pens in 4 three-tier batteries. Six unsexed chickens were housed per cage (28 cm × 48 cm × 48 cm) at the Poultry Research Centre, King Abdulaziz City for Science and Technology (KACST), Al- Muzahimiyah, Saudi Arabia. Each pen had a 1 cm squared wire mesh bottom for waste collection and was equipped with a feeding trough placed outside and two water cups inside the pen. The batteries were placed in a windowless room equipped with forced ventilation. The light was continuous during the experiment, and the temperature was gradually reduced from 33 to 23°C until the termination of the study. Veterinary care was provided, and vaccination programs were implemented according to the husbandry practice for broiler chickens; both were carried out under the supervision of a veterinarian.

### Measurements

Chick performance was measured in terms of feed consumption, body weight, feed conversion rate, and survival rate using the replicate as the experimental unit. Chickens were weighed at 1, 21, and 35 d of age before being offered the feeds and sexed at 35 days by comb size to correct for sex differences within replicates and among treatments. At 21 and 35 d of age, feed intake was calculated. The feed intake and body weight gain data were used for the calculation of FCR. Mortality was recorded daily, and the survival rate was calculated for the complete experimental period. The survival rate was the number of live broilers at 35 d of age divided by the number of 1 day-old broilers. The European production efficiency index (EPEI) was calculated according to (15).

After being fasted overnight, four males chickens from each treatment group, i.e., one chicken per replicate, were randomly slaughtered at 21 and 35 d of age according to the Islamic method (34, 35). The carcass, abdominal fat, breast muscles, unskinned right and left thighs, and thigh muscles were weighed and expressed in relation to living body weight. Besides, the gizzard, heart, liver, bursa, thymus, spleen, duodenum (pancreatic loop), jejunum (from the pancreatic loop to Meckel's diverticulum), ileum (from Meckel's diverticulum to the ileocecal junction), and ceca were separated, measured, weighed, and expressed in relation to body weight. The intestinal organs were cleaned before being weighed. The weight : length ratios of the 3 segments (duodenum, jejunum, and ileum) were calculated as indicators of intestinal density (36). All of the data regarding organ weight and length were expressed per 100 g of body weight.

### Statistical Analysis

The power analyses were run to estimate the number of replicates using 0.10 difference in FCR of broilers chickens at market age, i.e., 1.3 vs. 1.5 kg/kg, the standard division of 0.10 kg/kg, two sides test, P-value of 0.05 and the desired power of 80. The estimated number of replicates was 4 replicates according to [https://www.stat.ubc.ca/~rollin/stats/ssize/n2.html]. The differences in FCR was considered to be 0.2 based in published resulted by (2, 37, 38).

The data were analyzed using the SAS software package (39), SAS Institute, Cary, NC, the USA with two-way variance analysis. In preliminary statistical analyses, sex differences within treatments and replicates were tested and it was not significant. Thus, we run the statistical analyses based on the replicate for body weight gain, feed intake, FCR and European production index. The data were analyzed using the GLM procedure of SAS using the replicate as the experimental unit according to the following model:

$$\begin{array}{l} \text{Yij}=\text{m}+\text{Ti}+\text{Bj}+\text{Yij}+\text{eijk} \end{array}$$

Where: Y is a single observation, m is the general mean, Ti is the effect of dietary lupine concentrations, Bj is the effect of supplements, Yij is the interaction between lupine concentration and supplements, and eijk is the experimental error.

Before the analysis, all percentages were subjected to logarithmic transformation (log10 x + 1) to normalize data distribution. Mean differences were tested at *P* ≤ 0.05 by all possible differences (39). The data were presented based on mean and SEM.

## Results and Discussion

### Chemical Composition and Energy Value

The chemical composition of blue lupine was 92.5% dry matter (DM), 30.4% (CP), 5.39% fat, 2.51% ash, 16.2% crude fiber (CF), and 38.0% nitrogen-free extract (NFE). The published values for blue lupine are 35.5% CP, 5.45% fat, 16.5% CF, 4.01% ash, and 38.5% NFE (32). The calculated metabolizable energy value of the feed basis (92.5% DM) was 7.41 MJ/kg. The results of the present study showed that blue lupine might be a good source of nutrients such as lipids, fiber, minerals, and vitamins (10, 12). In addition, (40) found that white lupine beans contain 44% CP, 10.7% crude fat, 16.1% CF, 4.00% ash, and 13.9 MJ/kg of metabolizable energy. Moreover, narrow-leaved lupine and yellow lupine consist of 89.1 and 87.1% DM, 35.4, and 41.2% CP, 5.96, and 5.45% crude fat, 17.9 and 15.5% CF, 3.71 and 5.45% ash, and 37.1 and 31.1% (soluble carbohydrate) NFE, respectively (16). The differences between our values and those mentioned in the literature regarding the chemical composition of lupine can be attributed to the variety of lupine strains (10, 16).

In addition, lupine proteins are superior to and more degradable than proteins of other legumes, e.g., soybean (41, 42). Moreover, blue lupine seeds are rich in lysine (43). Mukisira (44) indicated that lupine protein has relatively high amounts of threonine, lysine, and tryptophan (45), but low methionine content. The sulfur-containing amino acids are primarily limited in lupine protein (46). However, the lupine amino acid balance compares positively with that of soybean (47). The superiority of blue lupine protein to soybean meal may be due to its lower phytic acid and saponin levels as well as its lower lectin and protease inhibitor concentrations (48).

### Growth Performance

The effects of the dietary addition of blue lupine and/or probiotics on growth performance are shown in Tables 3, 4. The blue lupine and/or probiotics did not significantly affect feed intake, body weight gain (Table 3), and the EPEI (Table 4) during different experimental periods or the FCR from 1 to 21 and 1 to 35 d of age. Nonetheless, the FCR of broilers that were fed the 30% blue lupine diet during the 22–35 d period was significantly impaired compared with the control group. This may be due to a low essential amino acids concentration, particularly that of arginine, lysine, threonine, tryptophan, and valine when blue lupine was fed at 30%. In addition, this may also be due to the high fiber content of this diet and thus, the influence of blue lupine on the gut ecosystem (18). Both the deficiency of essential amino acids and the higher fiber content can impair feed use for growth (21, 22). However, the FCR was similar among different blue lupine groups for the entire period we examined. Broilers showed improved tolerance to amino acid deficiency and high crude fiber concentration, especially the older ones (15), due to gut maturation (27). There was no significant difference in the FCR among broilers that were fed the 20% blue lupine diet and those that were fed the control or 30% blue lupine diets (Table 4). In the literature, blue lupine can be incorporated in broilers' diets in up to 25% of soybean meal protein and yield similar results to the soybean meal (16, 18) and white lupine diets (21, 22). Other researchers found that when blue lupine is fed at 30% of soybean meal protein (19, 40, 49) and 15% it does not have any negative influences on the production performance and health of layers (8), but increasing blue lupine to 40 and 80% of soybean meal protein decreased growth and impaired gut ecosystem and increased wet dropping (18, 19). Similar results were obtained with the inclusion of 25–30% of blue lupine in turkey diets (40, 49). In one study (2) it was observed that raw lupine fed at 40% and dehulled lupine seed meal fed at 35% significantly decreased the feed intake and growth of broiler chickens; this was visible during the first week of age and continued up to 21 d of age. These response differences to dietary lupine could be attributed to the type and dietary concentration of lupine and the negative impact of blue lupine on gut ecology (18, 19, 21, 50).

**Table 3:** Effect of blue lupine and probiotics on the growth performance parameters of broiler chicks at 1–35 d of age.

**Treatment**	**Feed intake 1-21 d of age, g/day**	**Feed intake 22–35 d of age, g/day**	**Feed intake 1-35 d of age, g/day**	**Initial body weight, g**	**Body weight gain 1–21 d of age, g**	**Body weight gain 22–35 d of age, g**	**Body weight gain 1–35 d of age, g**
**Effect of dietary blue lupine concentration**							
Control (0%)	1,039	1,779	2,817	43.1	773	1,132	1,941
20%	1,046	1,785	2,830	43.2	790	1,124	1,955
30%	1,039	1,833	2,872	43.2	772	1,123	1,942
**Effect of dietary probiotics**							
Unsupplemented (–)	1,020	1,778	2,798	43.1	769	1,110	1,922
Supplemented (+)	1,063	1,819	2,881	43.1	787	1,142	1,971
**Interaction between blue lupine concentration and probiotics**							
0% (–)	1,038	1,754	2,792	43.1	788	1,119	1,945
0 % (+)	1,039	1,804	2,843	43.1	758	1,145	1,937
20% (–)	1,011	1,746	2,757	43.2	767	1,095	1,904
20% (+)	1,081	1,823	2,904	43.2	813	1,152	2,007
30% (–)	1,011	1,835	2,847	43.2	752	1,116	1,916
30% (+)	1,068	1,830	2,898	43.2	791	1,130	1,968
***P*****-values**							
Blue lupine	0.97	0.57	0.71	0.35	0.70	0.97	0.95
Probiotic	0.13	0.38	0.16	0.99	0.36	0.37	0.22
Interaction	0.55	0.76	0.73	0.96	0.25	0.88	0.50
RMSE	65.5	110.5	137.0	0.10	48.3	86.1	93.6

**Table 4:** Effect of blue lupine and probiotics on feed conversion rate, European production efficiency index, and economic efficiency of broiler chickens from 1 to 35 d of age.

**Treatment**	**Feed conversion rate 1–21 d of age, g/day**	**Feed conversion rate 22–35 d of age, g /day**	**Feed conversion rate 1–35 d of age, g/day**	**Survival rate,%**	**European production efficiency index**
**Effect of dietary blue lupine concentration**					
Control (0%)	1.34	1.57^b^	1.45	100	382
20%	1.33	1.59^a^^b^	1.45	100	386
30%	1.35	1.64^a^	1.48	100	376
**Effect of dietary probiotics**					
Unsupplemented (–)	1.33	1.61	1.46	100	377
Supplemented (+)	1.35	1.60	1.46	100	385
**Interaction between blue lupine concentration and probiotic supplementation**					
0% (–)	1.32	1.57	1.44	100	388
0 % (+)	1.37	1.58	1.47	100	377
20% (–)	1.32	1.60	1.45	100	375
20% (+)	1.33	1.59	1.45	100	396
30% (–)	1.35	1.65	1.49	100	369
30% (+)	1.35	1.62	1.47	100	384
***P*****-values**					
Blue lupine	0.72	0.04	0.20	ND	0.69
Probiotic	0.44	0.58	0.72	ND	0.40
Interaction	0.72	0.73	0.37	ND	0.36
RMSE	0.06	0.06	0.03	ND	22.6

a,b * Means for each trait with different superscripts differ significantly at p < 0.05; ND, Not done*.

The addition of probiotics had no significant effect on the growth performance of broilers (growth rate, feed intake, FCR, and EPEI) during different experimental periods (Tables 3, 4). These results are in agreement with those reported by (23–25, 51–53) that indicate that the dietary supplementation of probiotics has insignificant influence on the performance of broilers during the grower, finisher, and the whole periods (1–35 d- of age).

No significant interaction between blue lupine and probiotics on the growth performance and EPEI was observed. These results indicate that blue lupine could be included in broiler diets up to 30%, with or without the addition of probiotics, and lead to production performance and EPEI similar to those of the control group. However, probiotic addition seemed to be beneficial, particularly when added to a 30% blue lupine diet, which exhibited higher crude fiber and low amino acid concentrations, causing an improvement of 2.71, 1.34, and 4.07% in growth rate, FCR, and EPEI, respectively. These results are similar to those reported by (24, 25, 35, 54, 55), who found that probiotics containing *Bacillus* spp. and *Saccharomyces boulardii* benefit the production performance of broilers from 1 to 28 d of age. The positive effect of probiotics could be attributed to their ability to modulate gut ecology toward beneficial microflora, thus improving gut health and absorption capacity, thereby improve productive performance (31, 54, 56). Even, the present results supported the previous studies in literature, the results of growth performance found herein may be confounded by the number of replicates (4 per treatment) and thus, further experiments were suggested.

### Carcass Traits of 21 and 35 D-Old Broilers

The effects of different levels of blue lupine and/or the addition of probiotics on the carcass characteristics of broilers at 21 and 35 d of age are shown in Table 5. The inclusion of 20% and 30% of blue lupine in broiler diets with or without the addition of probiotics at 21 d, had no significant effect on the dressing and breast muscle percentages. The inclusion of 20 and 30% of blue lupine in broiler diets at 35 d showed significant differences between the 20% and the 30% level (Table 5). The legs of 35 d-old broilers were not affected by dietary blue lupine and its interaction with probiotics (Table 5). Their breast muscle percentage tended to decrease (*P* = 0.07) with the inclusion of 30% of blue lupine in their diet. The decrease in breast muscle may be due to the low essential amino acid concentrations of the 30% blue lupine diet that is essential for muscle protein deposition.

**Table 5:** Effect of blue lupine and probiotics on carcass traits of broiler chickens at 21 and 35 d of age.

**Treatment**	**Dressing, %**		**Breast muscles, %**		**Thighs, %**		**Drum sticks,%**	**Legs, %**
**Age (d)**	**21**	**35**	**21**	**35**	**21**	**35**	**35**	**35**
**Effect of dietary blue lupine concentration**								
Control (0%)	57.2	63.1	26.7	25.1^a^	24.6	10.1	8.55	18.7
20%	58.3	62.8	28.2	25.1^a^	24.8	10.1	8.50	18.6
30%	57.4	63.1	28.3	23.4^b^	24.3	10.4	8.84	19.3
**Effect of dietary probiotics**								
Probiotic (–)	57.7	62.7	28.1	24.0	24.4	10.4	8.86^a^	19.2
Probiotic (+)	57.5	63.3	27.3	25.0	24.8	10.1	8.40^b^	18.5
**Interaction between blue lupine concentration and probiotics**								
0 (–)	57.7	62.8	27.8	24.6	24.2	10.4	8.74	19.1
0 (+)	56.8	63.5	25.6	25.5	25.0	9.86	8.36	18.2
20 (–)	58.1	62.4	28.4	24.4	24.6	10.1	8.86	19.0
20 (+)	58.4	63.1	27.9	25.7	25.0	10.1	8.14	18.3
30 (–)	57.5	62.9	28.1	23.0	24.3	10.6	8.98	19.6
30 (+)	57.3	63.4	28.5	23.7	24.4	10.2	8.70	18.9
***P*****-values**								
Blue lupine (A)	0.63	0.85	0.36	0.07	0.76	0.62	0.22	0.33
Probiotic (B)	0.81	0.32	0.43	0.15	0.40	0.31	0.02	0.07
Interaction A × B	0.88	0.98	0.56	0.93	0.83	0.72	0.54	0.98
RMSE	2.27	1.73	2.38	1.77	1.16	0.77	0.46	1.07

a,b *Means for each trait with different superscripts differ significantly at p < 0.05*.

There was no significant effect of the interaction between the blue lupine and probiotic addition on carcass traits at 21 and 35 d (Table 5). A similar trend was observed at 35 d of age with the exception of probiotic addition that significantly decreased the drumstick percentage compared to the unsupplemented control groups (Table 5). This drumstick percentage decrease (5.19%) concurred with numerical increases in the breast muscle percentage (4.17%). The present results demonstrated that blue lupine and probiotics had no adverse effect on carcass traits. These results are in agreement with those reported by (40) who observed that 14.0–25.7% of white lupine in broilers' and turkeys' diets has no adverse effect on slaughter yield and carcass quality. In addition, (49) noticed that 25–30% of blue lupine does not significantly affect the dressing percentage, edible parts, and abdominal fat of turkeys. On the other hand, (55) demonstrated that probiotics at 100 and 150 g/ton feed containing *Bacillus* spp. and *Saccharomyces boulardii*, reduced the dressing percentages, breast muscle values, liver weights, and abdominal fat weights; a 50 g/ton diet had no effect.

### Immune Organs at 21 and 35 D of Age

The inclusion of 20 and 30% of blue lupine had no significant effects on the bursa, thymus, and spleen percentages at 21 and 35 d of age (Table 6). These results indicated that blue lupine had no negative influence on lymphoid organs and immune index of broilers. This suggests that blue lupine diets without or with probiotics provide adequate nutrients for the growth of immune organs.

**Table 6:** Effect of blue lupine and probiotics on the internal body organs of broiler chickens at 21 and 35 d of age.

**Treatment**	**Bursa, %**		**Thymus, %**		**Spleen, %**	
**Age (d)**	**21**	**35**	**21**	**35**	**21**	**35**
**Effect of dietary blue lupine concentration**						
Control (0%)	0.16	0.17	0.37	0.43	0.084	0.110
20%	0.17	0.16	0.43	0.40	0.101	0.110
30%	0.16	0.16	0.38	0.49	0.086	0.130
**Effect of dietary probiotics**						
Probiotic (–)	0.16	0.15	0.41	0.38^b^	0.096	0.113
Probiotic (+)	0.17	0.17	0.37	0.49^a^	0.084	0.120
**Interaction between blue lupine concentration and probiotics**						
0 (–)	0.17	0.12^b^	0.43^a^^b^	0.33	0.078	0.100
0 (+)	0.14	0.21^a^	0.31^b^	0.53	0.090	0.120
20 (–)	0.14	0.16^a^^b^	0.53^a^	0.38	0.109	0.120
20 (+)	0.20	0.15^a^^b^	0.33^b^	0.42	0.092	0.100
30 (–)	0.17	0.16^a^^b^	0.28^b^	0.44	0.100	0.120
30 (+)	0.16	0.16^a^^b^	0.47^a^^b^	0.54	0.071	0.139
***P*****-values**						
Blue lupine (A)	0.82	0.90	0.60	0.28	0.17	0.21
Probiotic (B)	0.57	0.09	0.41	0.03	0.15	0.53
Interaction A × B	0.08	0.04	0.02	0.41	0.12	0.21
RMSE	0.04	0.04	0.13	0.13	0.02	0.03

a,b *Means for each trait with different superscripts differ significantly at p < 0.05*.

There was a significant effect of the interaction between the blue lupine and probiotics on the bursa percentage at 35 d of age, as well as the thymus percentage at 21 d of age (Table 6). At 21 d of age, the bursa percentage of broilers that were fed a 20% blue lupine diet had the highest increase due to probiotic addition, compared to probiotic-supplemented control groups and probiotic-unsupplemented 20% blue lupine groups; however, the increase did not reach a level of significance (*P* = 0.08). At 35 d of age, the bursa percentage of broilers that were fed the control diet supplemented with probiotics, significantly increased when compared to the unsupplemented control group. This trend was different from that observed at 21 d and indicated that the effect of probiotic addition on bursa measurements depends on the age of broilers and the level of blue lupine in their diet (Table 6).

Probiotic addition had no significant influence on the lymphoid organs percentage at 21 d of age (Table 6). The thymus percentage of 35 d-old broilers increased significantly (29.3%) in the probiotic-supplemented group, compared to the unsupplemented group. These results indicate that a prolonged probiotic feeding period until 35 d of age significantly increased the thymus percentage in the probiotic-supplemented group as compared to the unsupplemented group; however, these results are in contrast to what was obtained at 21 d of age. Probiotic addition to a 20% blue lupine diet significantly decreased the thymus percentage (0.33%) as compared to the effects of the same diet without the addition of probiotics on the thymus percentage (0.53%). In addition, a 30% blue lupine diet without the addition of probiotics, significantly decreased the thymus percentage (0.28%) compared to a 20% blue lupine diet without probiotic addition (0.53%). These results indicate that an unsupplemented, 30% blue lupine diet may have a negative effect on the thymus percentage. The addition of probiotics may have caused a complete recovery of the thymus percentage, suggesting a beneficial effect of probiotics on the lymphoid organs of broilers fed 30% blue lupine due to improving gut ecosystem and thus increasing nutrients available for growth of immune organs. Thus, 30% of blue lupine, supplemented with probiotics, could be included in broiler diets without any negative effects on immunity. It is well-known that thymus has an important role in the preparation and development of T-lymphocytes or T cells, extremely important leukocyte types (30). In general, probiotic addition had a beneficial effect on the bursa, thymus, and spleen percentages of broilers that were not fed blue lupine at 35 d of age. These results are similar to those reported by (55, 57), who found that the addition of probiotics improved the immune index of broiler chickens and beneficially modulated gut microflora, particularly in the ceca area and resulted in enhanced growth (23–25).

### Internal Body Organs at 21 and 35 D of Age

The 20 and 30% of blue lupine with the addition of probiotics and the interaction between the two variables did not significantly affect the percentages of the liver, heart, and abdominal fat at 35 d of age (Table 7). However, the gizzard percentage at 21 d was significantly reduced (14.5%) by the inclusion of 20% of lupine, compared to the control group. The inclusion of 20 and 30% of blue lupine in broiler diets increased the gizzard percentage by 16.8 and 21.1%, respectively, at 35 d of age. The increase in the gizzard observed at 35 d reflected the increase in the CF percentage of the tested diets and indicated that a prolonged feeding period might be needed for blue lupine to exert an effect.

**Table 7:** Effect of blue lupine and probiotics on internal body organs of broiler chickens at 21 and 35 d of age.

**Treatment**	**Liver, %**		**Heart, %**		**Gizzard, %**		**Abdominal fat**, **%**	
**Age (d)**	**21**	**35**	**21**	**35**	**21**	**35**	**21**	**35**
**Effect of dietary blue lupine concentration**								
Control (0%)	2.66	2.23	0.58	0.45	2.96^a^	1.61^b^	1.60^a^	1.17
20%	2.51	2.12	0.68	0.46	2.53^b^	1.88^a^	0.76^b^	1.31
30%	2.59	2.40	0.66	0.47	2.80^a^^b^	1.95^a^	0.59^b^	0.99
**Effect of dietary probiotics**								
Probiotic (–)	2.63	2.14	0.68	0.46	2.73	1.77	0.98	1.17
Probiotic (+)	2.55	2.29	0.60	0.46	2.80	1.86	0.98	1.14
**Interaction between blue lupine concentration and probiotics**								
0 (–)	2.72	2.29	0.59	0.43	2.84	1.63	1.59	1.18
0 (+)	2.61	2.17	0.57	0.46	3.08	1.59	1.62	1.17
20 (–)	2.49	2.02	0.74	0.48	2.57	1.87	0.59	1.29
20 (+)	2.53	2.21	0.62	0.45	2.49	1.87	0.93	1.34
30 (–)	2.68	2.11	0.72	0.47	2.76	1.81	0.77	1.05
30 (+)	2.50	2.48	0.59	0.47	2.84	2.09	0.40	0.93
***P*****-values**								
Blue lupine (A)	0.65	0.64	0.27	0.64	0.03	0.02	0.02	0.24
Probiotic (B)	0.54	0.35	0.11	0.99	0.56	0.24	0.99	0.85
Interaction A × B	0.80	0.43	0.59	0.50	0.59	0.20	0.40	0.91
RMSE	0.33	0.42	0.13	0.05	0.30	0.20	0.51	0.42

a,b *Means for each trait with different superscripts differ significantly at p < 0.05*.

The percentages of abdominal fat at 21 d of age (Table 7) significantly decreased due to feeding 20 and 30% blue lupine diets, by 52.3 and 63.5%, respectively, compared to the control, but this effect diminished at 35 d of age. These beneficial effects indicate that the meat of broilers fed blue lupine diets may be leaner and contain less fat; thus, maybe healthier (1). Similarly, (15, 21, 22) observed that 14.0–25.7% of white lupine in broiler and turkey diets improves the meat quality of carcasses, decreases saturated fatty acids and increases polyunsaturated n-3 fatty acids, thus, making the meat more nutritious and heather.

In general, our results indicate that blue lupine and probiotics had no adverse effects on the liver and the heart. In partial agreement with our results, other research has shown that the addition of 10% of lupine in broiler diets during the first 1–14 d of age and the addition of 15 or 25% from 15 to 35 d of age significantly increases the liver and gizzard weights but does not affect abdominal fat (16). Regarding the effect of probiotics, other authors have demonstrated that probiotics at 100 and 150 g/ton feed containing *Bacillus* spp. and *Saccharomyces boulardii* decrease liver weights and abdominal fat (31, 35, 55).

### Upper Intestinal Morphology at 21 and 35 D of Age

The effect of blue lupine with or without probiotics on duodenum morphology at 21 and 35 d of age are displayed in Tables 8, 9, respectively. Broilers fed a 30% blue lupine diet had a significantly increased duodenum percentage and length at 21 and 35 d of age, respectively, when compared to broilers fed the control diet and the 20% blue lupine diet. The duodenum weight percentage at 35 d of age, the duodenum length percentage at 21 d of age, and the duodenum weight/length ratio at 21 and 35 d of age were not significantly affected by the blue lupine. The duodenum percentage and length increase could be due to the NSP content in lupine, particularly in the groups on the 30% blue lupine diet. The duodenum is involved in feed digestion (16, 50, 58). Lupine has high CF and NSP contents (14). The main NSP in lupine seeds is galactan; this consists of different amounts of arabinose and galactose monosaccharides. NSPs impair gut ecology and reduce the digestibility of poultry diets (2, 6, 15, 16). Thus, pectinase supplementations to 10% lupine diet improve chicken' performance and eliminate anti-nutritional factors, thus enhance nutrient availability. The results of the present study are similar to those reported by (2), who observed that all sections of the intestinal canal as well as the duodenum, jejunum, ileum, and ceca lengths significantly increase in lupine fed groups compared to control groups; there were no effects on mucosa, submucosa, and serosa morphology.

**Table 8:** Effect of blue lupine, probiotics, and age of chickens on internal body organs of broiler chickens at 21 and 35 d of age.

**Treatment**	**Duodenum**, **%**		**Duodenum** **length, %**		**Duodenum** **weight/length**	
**Age (d)**	**21**	**35**	**21**	**35**	**21**	**35**
**Effect of dietary blue lupine concentration**						
Control (0%)	0.88^b^	0.53	3.02	1.29^b^	0.29	0.42
20%	0.95^b^	0.53	3.08	1.29^b^	0.31	0.41
30%	1.04^a^	0.57	3.39	1.50^a^	0.31	0.39
**Effect of dietary probiotics**						
Probiotic (–)	1.02^a^	0.55	3.32^a^	1.36	0.31	0.41
Probiotic (+)	0.89^b^	0.54	3.00^b^	1.36	0.30	0.40
**Interaction between blue lupine concentration and probiotics**						
0 (–)	0.97^b^	0.55	3.26	1.36	0.30	0.41
0 (+)	0.78^c^	0.51	2.79	1.23	0.28	0.42
20 (–)	0.94^b^	0.55	3.01	1.34	0.32	0.41
20 (+)	0.96^b^	0.51	3.15	1.24	0.31	0.41
30 (–)	1.15^a^	0.56	3.71	1.39	0.31	0.40
30 (+)	0.94^b^	0.59	3.08	1.60	0.31	0.38
***P*****-values**						
Blue lupine (A)	0.03	0.30	0.12	0.03	0.46	0.42
Probiotic (B)	0.01	0.55	0.04	0.93	0.58	0.71
Interaction A × B	0.02	0.38	0.10	0.09	0.93	0.69
RMSE	0.08	0.07	0.36	0.19	0.04	0.05

a,b,c *Means for each trait with different superscripts differ significantly at p < 0.05*.

**Table 9:** Effect of blue lupine and probiotics on jejunum and ileum measurements of broiler chickens at 21 and 35 d of age.

**6 Treatment**	**Jejunum, %**		**Jejunum length, %**		**Jejunum weight/length**		**Ileum, %**		**Ileum length, %**		**Ileum weight/length**	
**Age (d)**	**21**	**35**	**21**	**35**	**21**	**35**	**21**	**35**	**21**	**35**	**21**	**35**
**Effect of dietary blue lupine concentration**												
Control (0%)	2.10	1.71	8.05	3.42	0.26	0.50	1.66	1.50^b^	7.61	3.46	0.22	0.43^b^
20%	1.77	1.81	7.27	3.48	0.24	0.52	1.62	1.78^a^	7.31	3.46	0.23	0.52^a^
30%	2.07	1.76	7.57	3.72	0.27	0.47	1.90	1.67^a^^b^	7.15	3.62	0.28	0.46^b^
**Effect of dietary probiotics**												
Probiotic(–)	2.09	1.79	8.00^a^	3.60	0.26	0.50	1.65	1.64	7.73	3.57	0.22	0.46
Probiotic(+)	1.87	1.73	7.26^b^	3.48	0.26	0.50	1.81	1.66	6.99	3.46	0.27	0.48
**Interaction between blue lupine concentration and probiotics**												
0 (–)	2.13	1.62	8.34	3.49	0.25	0.47^a^^b^	1.54	1.43	8.07	3.45^a^^b^	0.20	0.41
0 (+)	2.08	1.80	7.76	3.34	0.27	0.54^a^	1.79	1.57	7.15	3.47^a^^b^	0.25	0.45
20 (–)	1.68	1.81	7.24	3.61	0.23	0.50^a^^b^	1.39	1.82	7.19	3.72^a^	0.20	0.49
20 (+)	1.87	1.81	7.29	3.35	0.25	0.54^a^	1.86	1.74	7.44	3.19^b^	0.25	0.55
30 (–)	2.47	1.94	8.43	3.69	0.29	0.53^a^	2.01	1.66	7.94	3.54^a^^b^	0.25	0.47
30 (+)	1.66	1.57	6.71	3.75	0.25	0.42^b^	1.79	1.70	6.36	3.70^a^	0.31	0.45
***P*****-values**												
Blue lupine (A)	0.31	0.76	0.18	0.23	0.47	0.34	0.44	0.05	0.68	0.42	0.19	0.02
Probiotic (B)	0.24	0.62	0.04	0.44	0.93	0.99	0.39	0.80	0.10	0.32	0.06	0.25
Interaction A × B	0.10	0.15	0.12	0.67	0.36	0.01	0.32	0.57	0.24	0.05	0.99	0.42
RMSE	0.45	0.31	0.81	0.40	0.05	0.07	0.45	0.24	1.06	0.31	0.07	0.06

a,b *Means for each trait with different superscripts differ significantly at p < 0.05*.

At 21 d of age, a significant reduction in duodenum percentage (12.8%) and duodenum length percentage (9.64%) was observed in chicks that received probiotics, compared to those that were on diets without probiotics (Table 8); this effect disappeared at 35 d of age (Table 8), suggesting that the effect of probiotics may depend on the age of chickens and be more prominent in early age (24, 25).

There was a significant reduction in the duodenum percentage at 21 d of age due to probiotic addition both in control and the 30% blue lupine diet group that amounted to 19.6 and 18.3%, respectively. Probiotic supplementation increased the duodenum length at 35 d of age in broilers that were fed the 30% blue lupine diet (Table 8). Meanwhile, there were no significant effects of the interaction between the blue lupine and probiotics on the duodenum length percentage and the duodenum weight/length ratio (Table 8).

Probiotics decreased the duodenum percentage and duodenum length percentage at 21 d of age; this effect depended on the blue lupine level, but it diminished at 35 d of age (Table 8). These results indicate a reduction in duodenum percentage at 21 d of age in control and 30% blue lupine groups in response to the probiotic addition in the broilers' diet. This temporary adaptive effect shown at 21 d of age may indicate an increase in the absorption surface area that had been diminished after feeding a blue lupine diet for a prolonged period of time (24, 25). These changes indicate that the effect of probiotics on the weight percentage and length percentage of the duodenum depends on the diet profile, age of broilers, and the measurement of the duodenum (6, 15, 23).

### Lower Intestinal Morphology at 21 and 35 D of Age

Table 9 shows the influence of the blue lupine and/or probiotics on the jejunum and ileum characteristics of broilers at 21 and 35 d of age, respectively. The results show a significant effect of probiotics on the jejunum length percentage, indicating a significant decrease (9.25%) when compared to groups that were not supplemented with probiotics (Table 9). However, the influence of probiotics on the jejunum length percentage at 21 d of age (Table 9) was diminished at 35 d of age (Table 9). These results indicate that the addition of blue lupine and probiotics had no adverse effects on the lower small intestine parts' (jejunum and ileum) morphology; an exception to this was the increase in the ileum percentage and the ileum weight/length ratio of the 20% blue lupine diet group observed at 35 d of age. This effect was not observed at 21 d of age; this indicates that prolonged feeding of a 20% blue lupine diet may exert an effect on the ileum length percentage and weight/length ratio.

There were significant effects of the interaction between blue lupine and probiotics on the jejunum weight/length ratio and ileum length at 35 d. These results indicate that probiotics may have decreased the jejunum weight/length ratio of the 30% blue lupine diet group and the ileum length percentage of the 20% blue lupine diet group.

An increase in the ileum length may reflect an adaptive effect in the absorption site in response to feeding blue lupine at a 20% rate. It is well-known that the lower small intestine is mainly involved in nutrient absorption (58). The results of the present study indicate that broiler chickens could be fed a diet containing up to 30% of blue lupine without adverse effects on their lower small intestine parts' (jejunum and ileum) morphology and function.

### Small Intestine Morphology and Cecal Morphology at 21 and 35 D of Age

Table 10 displays the influences of blue lupine and/or probiotics on the small intestine and ceca morphology at 21 and 35 d of age, respectively. At 21 d of age, the effect of blue lupine and probiotics was not significant (*P* < 0.05) on the small intestine percentage, small intestine weight /length ratio, and ceca weight/length ratio. However, the addition of probiotics significantly decreased the small intestine length percentage compared to the control group at 21 d of age. There was no significant influence of either the blue lupine or probiotics on the small intestine percentage or the ceca percentage, length, and weight/length ratio at 35 d of age.

**Table 10:** Effect of blue lupine and probiotics on small intestine parts of broiler chickens at 21 and 35 d of age.

**Treatment**	**Small intestine, %**		**Small intestine length, %**		**Small intestine, weight/length**		**Ceca weight, %**		**Ceca** **length,%**		**Ceca** **weight/length**	
**Age (d)**	**21**	**35**	**21**	**35**	**21**	**35**	**21**	**35**	**21**	**35**	**21**	**35**
**Effect of dietary blue lupine concentration**												
Control (0%)	4.65	3.75	18.7	8.17	0.25	0.46^b^	1.31	0.82	2.09	1.83	0.62	0.46
20%	4.35	4.11	17.7	8.22	0.26	0.50^a^	1.41	0.951	2.07	1.92	0.66	0.50
30%	5.01	3.99	18.1	8.84	0.28	0.46^b^	1.61	0.890	2.15	1.91	0.73	0.47
**Effect of dietary probiotics**												
Probiotic (–)	4.77	3.97	19.1^a^	8.53	0.25	0.47	1.45	0.878	2.17	1.85	0.65	0.48
Probiotic(+)	4.58	3.93	17.2^b^	8.29	0.27	0.48	1.43	0.892	2.03	1.92	0.69	0.47
**Interaction between blue lupine concentration and probiotics**												
0 (–)	4.65	3.62	19.7^a^^b^	8.30	0.24	0.44^b^^c^	1.16^b^	0.722	2.00^b^	1.72	0.56	0.45
0 (+)	4.65	3.88	17.7^b^^c^	8.04	0.26	0.48^a^^b^	1.47^a^^b^	0.908	2.18^a^^b^	1.93	0.67	0.47
20 (–)	4.03	4.16	17.5^b^^c^	8.66	0.23	0.48^a^^b^	1.30^b^	0.891	2.02^b^	1.87	0.61	0.48
20 (+)	4.68	4.06	17.9^abc^	7.78	0.26	0.52^a^	1.52^a^^b^	1.01	2.12^a^^b^	1.96	0.70	0.52
30 (–)	5.63	4.14	20.1^a^	8.62	0.28	0.48^a^^b^	1.90^a^	1.02	2.51^a^	1.96	0.77	0.53
30 (+)	4.40	3.84	16.2^c^	9.06	0.28	0.43^c^	1.31^b^	0.768	1.80^b^	1.87	0.70	0.41
***P*****-values**												
Blue lupine (A)	0.22	0.20	0.42	0.13	0.26	0.04	0.23	0.40	0.84	0.70	0.37	0.76
Probiotic (B)	0.53	0.77	0.01	0.42	0.26	0.55	0.88	0.87	0.25	0.45	0.51	0.68
Interaction A × B	0.06	0.37	0.04	0.19	0.75	0.02	0.03	0.06	0.02	0.45	0.50	0.33
RMSE	0.73	0.44	1.53	0.78	0.04	0.04	0.33	0.22	0.30	0.27	0.16	0.12

a,b *Means for each trait with different superscripts differ significantly at p < 0.05*.

There was a significant effect of the interaction between the blue lupine level and the probiotics on the small intestine length percentage at 21 d of age, the small intestine weight/length ratio at 35 d of age, and the ceca weight and length percentage at 21 d (Table 10).

At 21 d of age, the small intestine length percentage of broilers fed a 30% blue lupine diet without probiotics, increased significantly compared to that of broilers fed a 20% blue lupine diet without probiotics and the control group with probiotics, reflecting the CF content of the diet. At 35 d of age, the addition of probiotics to a 30% blue lupine diet significantly decreased the small intestine weight/length ratio compared to the other groups.

Generally, broilers offered a 30% blue lupine diet without probiotics, had significantly increased ceca weight and length percentages compared to groups on 0 and 20% blue lupine diets without probiotics. The addition of probiotics to a 30% blue lupine diet diminished the negative effect (enlargement) of blue lupine on the ceca weight and length percentages and the small intestine percentage. This reflected the CF content of the diet and the role of probiotics in improving gut ecology and health (23–25). The main function of ceca is water and electrolyte absorption and fermentation (58). Additionally, it is, to a very large extent, affected by diet, and it enlarges as a consequence of an increased amount of fermentable material, particularly fiber, in the diet (59). The beneficial effects of probiotic supplementation to a 30% blue lupine diet at 21 d of age are similar both in the ceca and the small intestine length percentages (Table 10). Previous research showed also similar results regarding the positive effect of probiotics on gut health (23–25).

## Conclusions

The addition of blue lupine (*L. Angustifolius*) seeds at a 30% rate in broiler diets supplemented with DL-methionine, L-lysine, L-threonine, and probiotics (*B. Subtilis*) could be a suitable protein source for broilers, without any adverse effects on growth performance, carcass characteristics, immune organs, and gut morphology of broilers. Probiotics also maintain gut enhanced gut health and immune organs and improved growth of broilers on 20 and 30% blue lupine diets. The limitation of this experiment is the small number of replicates (4) which may confound the growth performance results and its application, and thus the number of replicates could be increased in the further experiment.

## Data Availability Statement

The datasets generated for this study are available on request to the corresponding author.

## Ethics Statement

The experimental procedures were approved by King Abdulaziz City for Science and Technology, Riyadh, Saudi Arabia that recommends animal rights, welfare and minimal stress and did not cause any harm or suffering to animals according to the Royal Decree number M59 in 14/9/1431H.

## Author Contributions

All authors listed have made a substantial, direct and intellectual contribution to the work, and approved it for publication.

### Conflict of Interest

The authors declare that the research was conducted in the absence of any commercial or financial relationships that could be construed as a potential conflict of interest.

## Abbreviations

FCR: feed conversion rate
AMEn: nitrogen corrected metabolizable energy
CP: Crude protein
NFE: Nitrogen free extract (starch + sugar)
KACST: King Abdulaziz City for Science and Technology
d: days
EPEI: European production efficiency index
SAS: statistical analyses software
GLM: general linear model
SEM: standard error or mean
CF: crude fiber
CFU: colony-forming unit
NSP: non-starch polysaccharide.
